# The Relationship of Physical Activity and Dietary Quality and Diabetes Prevalence in US Adults: Findings from NHANES 2011–2018

**DOI:** 10.3390/nu14163324

**Published:** 2022-08-13

**Authors:** Furong Xu, Jacob E. Earp, Alessandra Adami, Lee Weidauer, Geoffrey W. Greene

**Affiliations:** 1 School of Education, University of Rhode Island, Kingston, RI 02881, USA; 2 Department of Kinesiology, University of Connecticut, Storrs, CT 06269, USA; 3 Department of Kinesiology, University of Rhode Island, Kingston, RI 02881, USA; 4 School of Health and Consumer Sciences, South Dakota State University, Brookings, SD 57007, USA; 5 Department of Nutrition and Food Sciences, University of Rhode Island, Kingston, RI 02881, USA

**Keywords:** diabetes mellitus, physical activity, diet, young adults, middle-aged adults, older adults

## Abstract

This study aimed to examine the relationship of physical activity and/or dietary quality and diabetes prevalence in the general population and within specific age groups. It was a cross-sectional study using 2011–2018 National Health and Nutrition Examination Survey and the US Department of Agriculture’s Food Patterns Equivalents data (*n* = 15,674). Physical activity was measured by Global Physical Activity questionnaire; dietary quality was analyzed using the Healthy Eating Index 2015; diabetes prevalence was determined by reported diagnosis and glycohemoglobin or fasting glucose. Data were analyzed using multiple logistic regression adjusted for demographic variables and weight status. Results revealed that although no statistically significant or non-substantial relationships were observed between dietary quality or physical activity and diabetes prevalence, respondents who did not meet physical activity recommendations regardless of dietary quality had a higher odds of diabetes prevalence than those who met physical activity recommendations and had a higher dietary quality (*p* < 0.05). In conclusion, meeting physical activity recommendations is an important protective factor for diabetes especially in combination with a higher quality diet. A healthy lifestyle appears to have the greater impact on diabetes prevention in middle-aged men and women.

## 1. Introduction

In the United States the annual healthcare costs related to diabetes are estimated to be around $327 billion, with the majority of these costs accrued among the 30.7–32.4 million US adults with type II diabetes (T2D) [1,2]. Diabetes prevalence increases exponentially with age, starting at 4.2% of younger adults (18–44 years) and reaching 17.5% and 26.8% in middle-aged adults (45–64 years) and older adults (65+), respectively [2]. Alarmingly, the incidence of diabetes has been increasing over time, with more than 90% of cases identified as T2D [2]. This trend is associated with a concomitant rise of healthcare costs that is expected to continue [2]. The growing prevalence of T2D has been attributed to the worsening obesity epidemic, as weight status has been identified as the most influential modifiable risk factor for the development of T2D [3,4]. However, given that some uncontrollable (e.g., sex, age, family history) and modifiable (e.g., physical activity and dietary quality) determinants of obesity risk are likewise determinants of T2D, it is important to understand the effects of modifiable risk factors, such as physical activity and diet quality, on T2D risk when accounting for weight status [4,5,6,7]. This knowledge can then be used to guide lifestyle modifications to reduce disease risk and improve overall health.

Specifically, as one of the important modifiable factors associated with diabetes, physical activity plays a direct role in controlling glycemic levels among people with T2D [8,9] by increasing blood glucose uptake and insulin sensitivity [10]. A meta-analysis confirmed the beneficial effects of physical activity interventions on glycemic control measured by glycohemoglobin in non-diabetic populations [11]. However, to the authors’ knowledge, only one study examined the relationship between physical activity and diabetes prevalence in the general adult population [12]. That study found higher physical activity levels were associated with a lower prevalence of diabetes, with a steeper reduction when the daily activity levels were lower [12]. However, they did not account for weight status or differentiate between age groups, thus further studies are warranted to examine the relationship of physical activity and diabetes considering these aspects.

A second modifiable factor associated with diabetes is dietary quality [13,14], defined by the US Health and Human Services and the US Department of Agriculture as compliance with dietary guidelines, quantified in the current study using the Healthy Eating Index (HEI) [15]. Studies focusing on food groups that are components of the HEI-2015 have found that higher dairy [16] or fruit and vegetable consumption [17] is associated with reduced risk of T2D. However, studies directly investigating the relationship between overall dietary quality and diabetes prevalence reported inconsistent results [18,19,20,21,22,23]. In fact, De Koning et al. found that higher dietary quality was inversely associated with the prevalence of T2D in men [18,19] and post-menopausal women [20,21], while other studies found no statistically significant relationship between dietary quality and the prevalence of diabetes in adults [22,23]. Additionally, while lifestyle interventions targeted at preventing diabetes commonly include both dietary and exercise interventions, thus far no studies have investigated the summative effects of following physical activity recommendations. Given that aging results in a number of physiological and functional changes to the body that can influence an individual’s ability to perform and respond to specific lifestyle modification [24], lifestyle differences in preventing diabetes that are most effective for each specific population group need to be investigated.

Given the arguments above, further research is warranted to investigate the relationship between physical activity and/or diet quality and diabetes prevalence in a representative sample of adults. This information is crucial for diabetes prevention and has important public health implications on this chronic condition. Therefore, the aim of the present study was to examine the association between leisure or recreational physical activity and dietary quality and diabetes prevalence using a nationally representative sample of the US adult population.

## 2. Methods

This study is a secondary data analysis using data from the National Health and Nutrition Examination Survey and the US Department of Agriculture’s Food Patterns Equivalents from 2011 to 2018 (*n* = 39,156) [25,26]. Exclusion criteria were: (1) subjects under 18 years of age (*n* = 15,331), (2) subjects 18 years or older but without a reported body mass index measure (*n* = 1357), have body mass index reported as underweight (*n* = 423), and do not have physical activity, two 24-h dietary recall data or an answer for diagnosed diabetes question (yes vs. no) (*n* = 5658). Furthermore, subjects with missing value for either fasting glucose or glycohemoglobin or pregnant women were also excluded (*n* = 713). As a result, a total of 15,674 respondents were allocated to the final analysis.

### 2.1. Diabetes

Diabetes for the present study was determined by (1) diagnosed diabetes: respondents reported diabetes diagnosis by doctor or health professional, or (2) undiagnosed diabetes: utilizing respondents glycohemoglobin and fasting glucose according to the Centers for Disease Control and Prevention’s (CDC’s) undiagnosed diabetes definition of either (a) glycohemoglobin test 6.5% or above, or (b) fasting glucose 126 mg/dL or more [2].

### 2.2. Physical Activity

Physical activity was assessed using weekly physical activity participation information collected by the Global Physical Activity questionnaire which was created by the World Health Organization [25,27]. Physical activity data were analyzed following the World Health Organization analysis guide [27]. Physical activity was converted to metabolic equivalent (MET) minutes of moderate to vigorous physical activity per week [27]. Respondents were classified based on criterion of having met (≥600 MET-minutes/week equivalent to 150 min/week of moderate intensity or 75 min/week vigorous intensity physical activity) or not meet (<600 MET-minutes/week) recommendations according to the physical activity guidelines for adults [28].

### 2.3. Diet Quality

Diet quality was analyzed using HEI-2015 scoring algorithm developed by the National Cancer Institute and the United States Department of Agriculture. HEI-2015 aligns with 2015–2020 Dietary Guidelines for Americans [15]. This analysis utilized the combined data from National Health and Nutrition Examination Survey’s two 24-h dietary recall and US Department of Agriculture’s Food Patterns Equivalents dataset [25,26]. The HEI-2015 includes 13 components. The maximum score for each component ranged from 5 to 10 adding up to a maximum score of 100 points [15]. Based on their HEI-2015 score tertile distribution, respondents were classified as: (1) higher dietary quality (the highest tertile, 60.1 < HEI-2015 ≤ 99.6) or (2) lower dietary quality (first two tertiles, 10 ≤ HEI-2015 ≤ 60.1).

### 2.4. Lifestyle Groups

Respondents were further grouped into four lifestyle groups based on whether or not they met physical activity recommendations and whether they had higher or lower dietary quality [29]. These lifestyle groups were (1) met physical activity recommendations + higher dietary quality, (2) met physical activity recommendations + lower dietary quality, (3) did not meet physical activity recommendations + higher dietary quality, and (4) did not meet physical activity recommendation + lower diet quality.

### 2.5. Demographics

The demographic characteristics for the present study included age (18–44 years, 45–64 years, 65 years or above), race/ethnicity (White, Black, Hispanic or others), education (high school or less, some college or above), and poverty income ratio (below, at or above poverty level) [25]. Body mass index was calculated using measured height and weight and further classified into the following weight status categories: (1) normal: 18.5 to <25, (2) overweight: 25 to <30, (3) obese: 30 or higher [25,30].

### 2.6. Data Analysis

The combined eight-year sample weight (dietary two-day sample weight) was used as the sample weight while conducting all analyses to calculate the estimate and its standard error. A design-based method was also used to estimate the standard error. For the respondents’ demographic characteristics, continuous variables are presented as weighted mean ± standard errors. The *p*-values were obtained by performing *t*-test (PROC SURVEYREG in SAS) and multiple comparisons were performed with Bonferroni correction. Whereas categorical variables are presented as count (weighted percentage) and their *p*-value was obtained by performing Chi-square test (PROC SURVEYFREQ in SAS). The odds ratio and *p*-value for the relationship between physical activity, diet quality and lifestyle groups and diabetes prevalence were obtained by performing multiple logistic regression (PROC SURVEYLOGISTIC) adjusted for sex, age, race/ethnicity, education, poverty income ratio and weight status. All the analyses were conducted using Statistical Analysis Software 9.4 (SAS Institute Inc., Cary, NC, USA) with *p* < 0.05 as the statistically significant level.

## 3. Results

Of 15,674 respondents, about half were females (50.9%), 34.5% were racial and ethnic minorities, 34.5% had a high school education or less, 13.9% were below the federal poverty level, 32.5% were overweight and 39.6% were obese (Table 1). Moreover, 63.5% met the physical activity recommendations and 31.8% had higher dietary quality. Thirteen percent of respondents had diabetes, with 10.2% reporting having diagnosed diabetes and 2.8% classified as having diabetes according to the CDC’s definition of undiagnosed diabetes (Table 1). Furthermore, among respondents who did not meet the physical activity recommendations and had a lower quality diet, 17.8% of the total (21.3% of males and 15.5% of females) had diabetes. In contrast, 10% of respondents overall (13.2% of males and 7.3% of females) had diabetes in the group categorized as meeting physical activity recommendations and having higher dietary quality. There were variations in the prevalence of diabetes between lifestyle groups (Table 2).

For physical activity and diabetes, the multiple analysis showed a statistically significant inverse but not substantial relationship between physical activity and diabetes prevalence (Table 3). More specifically, for every 100 MET-minutes/week physical activity time increase, the likelihood of having diabetes decreased by 1% (OR = 0.99, 95% CI: 0.99, 1.00). The present study also found a negative but non substantial relationship between physical activity and diabetes prevalence in middle-aged adults (OR = 0.99, 95% CI: 0.99, 1.00) and older adults (OR = 0.99, 95% CI: 0.99, 1.00). Similar patterns were also observed among males and females, respectively (Table 3). For dietary quality and diabetes prevalence, no statistically significant relationships were observed.

While comparing differences in diabetes prevalence between lifestyle groups, we observed a significant difference between lifestyle groups in all adults and in middle-aged respondents. More specifically, there were significant group differences among respondents who did not meet physical activity recommendations and had higher dietary quality (OR = 1.60, 95% CI: 1.20, 2.14) and who did not meet the physical activity recommendation and had lower dietary quality (OR = 1.36, 95% CI: 1.09, 1.69) compared to those who met physical activity recommendations and had higher dietary quality. Moreover, age-specific analyses showed that in middle-aged adults, a higher diabetes incidence was observed among those who did not meet the physical activity recommendations and had higher dietary quality (OR = 1.88, 95% CI: 1.29, 2.74), as well as among those who did not meet the physical activity recommendations and had lower dietary quality (OR = 1.57, 95% CI: 1.17, 2.09) compared to those who met physical activity recommendations and had higher dietary quality (Table 3). Similar patterns were observed in both male and female individuals (Table 3).

## 4. Discussion

In the present study we found that although there was an inverse but not substantial relationship between physical activity and diabetes prevalence when controlling for weight status, the physical activity level was related to the prevalence of diabetes among US adults. That is, there were higher odds of having diabetes in respondents who did not meet the physical activity recommendations regardless of dietary quality in comparison to those who met physical activity recommendations and had a higher dietary quality. These results highlight the importance of physical activity for the prevention of T2D independent of its role in obesity prevention and emphasize its importance in specific age groups. While this study did not find a relationship between dietary quality and diabetes, our findings add a key novel element on the relationships between physical activity and/or dietary quality and diabetes prevalence across different age groups, while independently accounting for weight status, using a nationally representative adult sample. Our results have meaningful public health implications given the physical and economic burden of diabetes [1,31].

The Inverse but not substantial relationship we found between physical activity and diabetes prevalence among adults in general was partially in support of what was previously discussed in Zhao and colleagues’ study [12]. Zhao et al. reported that adults had greater odds of having diabetes among those with a physical activity level at or below 2000 METs, but the relationship between physical activity and diabetes prevalence was overall nonlinear [12]. Direct comparisons between Zhao and our study are not possible given the different confounding factors, exclusion criteria and age range used [12]. When adjusted for weight status, the current study showed that the prevalence of diabetes within the representative US adult cohort was consistent with the most recent CDC report [2]. Although the increasing prevalence of T2D with age is well established [2], the increases over time are often attributed to the higher prevalence of obesity that occurs with aging. While the present study included a statistical adjustment to account for the independent influence of weight status, such adjustment did not account for the altered body composition that often occurs with aging, i.e., muscle mass decreases in an inconsistent manner compared to the body mass or fat mass changes [24]. The significant but not substantial relationship between physical activity and diabetes prevalence indicates that physical activity level might be important to lower the odds of diabetes risk. This has been further verified by our findings on higher diabetes prevalence among those respondents who did not meet physical activity recommendations. A possible explanation for the reduction of the odds of T2D through different levels of physical activity (e.g., 600 or more MET-minutes/week) is that the activity helps improves insulin sensitivity in skeletal muscles [32] and overall mitochondrial function [33]. If this is true, it is plausible that physical activity is less effective in older adults due to presence of mitochondrial dysfunction that is associated with the normal aging process [34]. Although these considerations may explain why meeting physical activity guidelines was not associated with a lower T2D prevalence, they could not support explanation of the inverse relationship seen between the physical activity and T2D risk in older women but not older men.

There were no statistically significant relationships observed between dietary quality and diabetes prevalence. This finding is supported by previous studies [22,23], but is inconsistent with others who reported an inverse relationship between dietary quality and diabetes incidence [18,19,20,21]. An explanation for our findings is that the majority of the participants with diabetes (~77%) reported having been diagnosed with diabetes. The CDC National Diabetes Statistics Report 2020 found that among US adults with diagnosed diabetes 77.8% reported having at least one usual source of diabetes care, such as a doctor or other healthcare professional [1]. Following diabetes treatment guidelines [35], these professionals would have provided dietary advice. A recent review found that dietary education was effective in improving diabetes control [36] suggesting that dietary education provided by healthcare providers may have been effective in improving dietary quality in this study. Because the current study is cross-sectional, it is impossible to tell whether the dietary quality changed after diagnosis. Therefore, it is important to address overall dietary quality for diabetes prevention and management, and future prospective studies considering overall dietary quality and diabetes are warranted.

A novelty of the present study is the examination of the prevalence of diabetes between different lifestyle groups while accounting for body mass index classification and comparing between age groups. Our primary findings showed that middle-aged adults who did not meet the physical activity recommendations had higher odds of diabetes in comparison with those who met the physical activity recommendations. No such differences were observed in younger adults (18–44 years) or older adults (65 years or older). These results suggest that simply meeting the physical activity guidelines may not associate with a decrease in T2D prevalence, or maybe this latter is overshadowed by genetic factors. As such, physical activity interventions to prevent T2D should not be built around the physical activity recommendations but rather should be part of an overall weight management strategy (younger adults) or viewed in a dose dependent manner (older adults). Additionally, despite the finding that dietary quality was not independently related to T2D prevalence, there may be a beneficial effect of combining physical activity and dietary interventions. The lowest prevalence was found in those individuals meeting the physical activity recommendations and having a higher quality diet. This finding adds to the literature and further highlights the importance of a healthy lifestyle in diabetes prevention and intervention in adults.

### Strengths and Limitations

To the authors’ knowledge, this is the first study to examine the prevalence of diabetes between different lifestyle groups, as well as the first to examine the individual and summative effects of physical activity and diet between different age groups using a nationally representative sample of US adults. Strengths include the clear definition of undiagnosed diabetes following CDC recommendations, a rigorous assessment of the diet and use of the HEI-2015 to define dietary quality, and a definition of meeting physical activity recommendations. Limitations include the self-report nature of physical activity assessment and the cross-sectional nature of the study. These preclude exploring the casualty of the relationship between diabetes prevalence and physical activity, as well as dietary quality. However, the physical activity assessment instrument is widely used for population-based data collection [27].

Moreover, when interpreting the results of the dietary analysis, it is important to understand the limitations of using the HEI-2015 as a tool to assess diet quality. The strength of the HEI-2015 is in providing a single measure on how closely a person follows dietary recommendations set by the US Health and Human Services and the US Department of Agriculture. This tool positively scores for higher consumption of nine key dietary components (total fruit, whole fruits, total vegetables, greens/beans, whole grains, dairy, total protein foods, seafood/plant protein and healthful fatty acids) and negatively scores four components (refined grains, sodium, added sugars and saturated fats). However, this tool assesses the entire diet in a weighted manner, as such no single component makes up more than 10% of the overall score. Therefore, while previous studies have found that higher dairy consumption [16] and fruit and vegetable consumption [17] reduce T2D risk, increasing consumption of one of these components may not significantly change a person’s HEI-2015. Additionally, the HEI-2015 does not account for the dietary quantity, thus, a higher score could theoretically be derived from a higher overall caloric consumption [15].

## 5. Conclusions

The main finding of the present study is the negative but non-substantial relationship between physical activity and the prevalence of diabetes. There were no statistically significant relationships between dietary quality and diabetes prevalence. However, respondents who did not meet the physical activity recommendations regardless of dietary quality had a higher odds of diabetes prevalence in comparison to those who met physical activity recommendations and had a higher dietary quality. This indicates the importance of meeting physical activity recommendations and dietary quality guidelines for diabetes prevention.

## Figures and Tables

**Table 1:** Demographic characteristics stratified by sex (*n* = 15,674).

Variable	Total	Male	Female	*p*-Value
	*n* = 15,674	*n* = 7579 (49.1%)	*n* = 8095 (50.9%)	
Age, *n* (weighted %)				
18–44 years	6522 (43.9)	3207 (46.1)	3315 (41.7)	<0.001 *
45–64 years	5128 (34.4)	2414 (33.7)	2714 (35.0)	0.233
≥65 years	3512 (18.7)	1723 (17.2)	1789 (20.2)	<0.001 *
Race/ethnicity, *n* (weighted %)				
White	6160 (65.5)	3030 (66.0)	3130 (65.1)	0.28
Black	3559 (10.9)	1674 (10.1)	1885 (11.6)	<0.001 *
Hispanic	3698 (15.0)	1706 (15.1)	1992 (14.9)	0.725
Others	2257 (8.5)	1169 (8.7)	1088 (8.4)	0.495
Education, *n* (weighted %)				
High school or less	6162 (34.5)	3117 (35.7)	3045 (33.3)	0.023 *
Some college or more	8752 (65.5)	4079 (64.3)	4673 (66.7)	0.023 *
Poverty income ratio, *n* (weighted %)				
<1.0	2970 (13.9)	1284 (12.4)	1686 (15.5)	<0.001 *
≥1.0	11,407 (86.1)	5660 (87.6)	5747 (84.5)	<0.001 *
Body Mass Index (kg/m^2^)	29.44 ± 0.13	29.16 ± 0.15	29.70 ± 0.16	0.003 *
Weight status, *n* (weighted %)				
Normal	4180 (27.3)	1957 (24.5)	2223 (30.0)	<0.001 *
Overweight	5033 (32.5)	2809 (37.1)	2224 (28.0)	<0.001 *
Obese	6370 (39.6)	2768 (37.9)	3602 (41.3)	0.013 *
Total diabetes, *n* (weighted %)	2773 (13.0)	1460 (14.2)	1313 (11.9)	0.005 *
Diagnosed diabetes, *n* (weighted %)	2128 (10.2)	1128 (11.2)	1000 (9.2)	0.004 *
Undiagnosed diabetes, *n* (weighted %)	645 (2.8)	332 (3.0)	313 (2.7)	0.496
Dietary quality				
Total diet quality score (HEI-2015)	53.76 ± 0.31	52.33 ± 0.32	55.13 ± 0.39	<0.001 *
1st tertile (10–47.6), *n* (weighted %)	5381 (34.3)	2829 (37.7)	2552 (31.1)	<0.001 *
2nd tertile (47.6–60.1), *n* (weighted %)	5213 (33.8)	2538 (34.3)	2675 (33.4)	0.408
3rd tertile (60.1–99.6), *n* (weighted %)	5080 (31.8)	2212 (28.0)	2868 (35.5)	<0.001 *
Total PA MET-minutes/week	3253.22 ± 81.88	4286.61 ± 148.30	2257.55 ± 68.95	<0.001 *
Met PA, *n* (weighted %) ^#^	9171 (63.5)	5011 (70.4)	4160 (56.8)	<0.001 *
PA + Diet. Qual., *n* (weighted %)				
Met PA + Higher Diet. Qual.^@^	3110 (21.7)	1488 (20.3)	1622 (23.1)	0.004 *
Met PA + Lower Diet. Qual.^&^	6061 (41.7)	3523 (50.1)	2538 (33.7)	<0.001 *
Not meet PA + Higher Diet. Qual.	1970 (10.1)	724 (7.7)	1246 (12.3)	<0.001 *
Not meet PA + Lower Diet. Qual.	4533 (26.5)	1844 (21.9)	2689 (30.9)	<0.001 *

Note: Data are present as weighted mean ± SE unless otherwise specified. Male and female values have been compared, *p*-values for continuous variables were obtained by performing *t*-test (PROC SURVEYREG), and *p*-values for categorical variables were obtained by performing Chi-square test (PROC SURVEYFREQ). HEI = Healthy Eating Index; PA = physical activity; MET = metabolic equivalent of task. ^#^ Weekly physical activity time ≥ 600 MET-minutes/week; ^@^ Higher dietary quality was defined as 60.1 < HEI-2015 ≤ 99.6 (3rd tertile); ^&^ Lower dietary quality was defined as 10 ≤ HEI-2015 ≤ 60.1 (1st and 2nd tertiles); * statistically significant difference between male and female.

**Table 2:** The prevalence of diabetes according to the four lifestyle groups (*n* = 15,674).

Variable	Group 1: Met PA ^#^ + Higher Diet. Qual.^@^	Group 2: Met PA + Lower Diet. Qual. ^&^	Group 3: Not Meet PA + Higher Diet. Qual.	Group 4: Not Meet PA + Lower Diet. Qual.
**Overall**				
Total diabetes	419 (10.0) ^b,c^	768 (9.6) ^d,e^	538 (21.3) ^b,d^	1048 (17.8) ^c,e^
Diagnosed	321 (8.0) ^b,c^	574 (7.3) ^d,e^	424 (17.4) ^b,d^	809 (13.8) ^c,e^
Undiagnosed	98 (2.0) ^b,c^	194 (2.3) ^d,e^	114 (3.9) ^b,d^	239 (4.0) ^c,e^
**Male**				
Total diabetes	257 (13.2) ^b,c^	468 (9.8) ^d,e^	232 (25.0) ^b,d^	503 (21.3) ^c,e^
Diagnosed	206 (11.1) ^a,b,c^	339 (7.4) ^a,d,e^	189 (20.7) ^b,d^	394 (16.6) ^c,e^
Undiagnosed	51 (2.1) ^c^	129 (2.4) ^d,e^	43 (4.3) ^d^	109 (4.7) ^c,e^
**Female**				
Total diabetes	162 (7.3) ^b,c^	300 (9.2) ^d,e^	306 (19.1) ^b,d,f^	545 (15.5) ^c,e,f^
Diagnosed	115 (5.5) ^b,c^	235 (7.0) ^d,e^	235 (15.4) ^b,d^	415 (11.9) ^c,e^
Undiagnosed	47 (1.8) ^b,c^	65 (2.1) ^d,e^	71 (3.7) ^b,d^	130 (3.6) ^c,e^

Note: Data are presented as *n* (weighted %). Post hoc multiple lifestyle group comparisons were performed with Bonferroni correction. PA = physical activity; MET = metabolic equivalent of task; HEI = Healthy Eating Index. ^#^ Met PA recommendations was defined as weekly physical activity time ≥ 600 MET-minutes/week; ^@^ Higher dietary quality was defined as 60.1 < HEI-2015 ≤ 99.6 (3rd tertile); ^&^ Lower dietary quality was defined as defined as 10 ≤ HEI-2015 ≤ 60.1 (1st and 2nd tertiles). ^a^ Group 1 different from Group 2 with a *p* < 0.05; ^b^ Group 1 different from Group 3 with a *p* < 0.05; ^c^ Group 1 different from Group 4 with a *p* < 0.05; ^d^ Group 2 different from Group 3 with a *p* < 0.05; ^e^ Group 2 different from Group 4 with a *p* < 0.05; ^f^ Group 3 different from Group 4 with a *p* < 0.05.

**Table 3:** Association between PA and/or dietary quality and diabetes prevalence.

	Total (*n* = 15,674)		Male (*n* = 7579)		Female (*n* = 8095)	
Variable	OR (95% CI)	*p*-Value	OR (95% CI)	*p*-Value	OR (95% CI)	*p*-Value
**Overall**						
PA or Diet. Qual.						
Total PA MET-minutes/week—per increase of 100 point	0.99 (0.99, 1.00)	<0.001 *	0.99 (0.99, 1.00)	0.021 *	0.99 (0.99, 1.00)	<0.001 *
Diet. Qual. (HEI-2015)—per increase of 10 point	1.02 (0.96, 1.08)	0.516	1.03 (0.95, 1.12)	0.446	1.00 (0.92, 1.09)	0.949
PA + Diet. Qual.						
Met PA + Higher Diet. Qual.	Ref	-	Ref	-	Ref	-
Met PA + Lower Diet. Qual.	1.00 (0.80, 1.24)	0.997	0.92 (0.67, 1.28)	0.62	1.18 (0.81, 1.70)	0.377
Not meet PA + Higher Diet. Qual.	1.60 (1.20, 2.14)	0.001 *	1.70 (1.14, 2.54)	0.008 *	1.61 (1.09, 2.38)	0.014 *
Not meet PA + Lower Diet. Qual.	1.36 (1.09, 1.69)	0.005 *	1.41 (1.02, 1.94)	0.036 *	1.35 (0.97, 1.88)	0.067
**Adults aged 18–44 years**						
PA or Diet. Qual.						
Total PA MET-minutes/week—per increase of 100 point	1.00 (1.00, 1.01)	0.288	1.00 (0.99, 1.00)	0.431	1.00 (0.99, 1.00)	0.386
Diet. Qual. (HEI-2015)—per increase of 10 point	0.95 (0.83, 1.09)	0.467	1.04 (0.86, 1.26)	0.657	0.90 (0.74, 1.08)	0.246
PA + Diet. Qual.						
Met PA + Higher Diet. Qual.	Ref	-	Ref	-	Ref	-
Met PA + Lower Diet. Qual.	1.29 (0.77, 2.18)	0.323	0.85 (0.38, 1.92)	0.694	1.94 (0.89, 4.24)	0.091
Not meet PA + Higher Diet. Qual.	1.93 (0.91, 4.10)	0.082	1.93 (0.64, 5.81)	0.233	2.26 (0.83, 6.16)	0.105
Not meet PA + Lower Diet. Qual.	1.45 (0.82, 2.57)	0.192	1.22 (0.44, 3.40)	0.695	1.73 (0.76, 3.94)	0.181
**Adults aged 45–64 years**						
PA or Diet. Qual.						
Total PA MET-minutes/week—per increase of 100 point	0.99 (0.99, 1.00)	<0.001 *	0.99 (0.99, 1.00)	0.010 *	0.99 (0.98, 1.00)	0.015 *
Diet. Qual. (HEI-2015)—per increase of 10 point	1.04 (0.96, 1.12)	0.377	1.04 (0.93, 1.18)	0.48	1.03 (0.91, 1.16)	0.66
PA + Diet. Qual.						
Met PA + Higher Diet. Qual.	Ref	-	Ref	-	Ref	-
Met PA + Lower Diet. Qual.	1.02 (0.73, 1.41)	0.908	0.91 (0.60, 1.39)	0.658	1.25 (0.68, 2.29)	0.461
Not meet PA + Higher Diet. Qual.	1.88 (1.29, 2.74)	<0.001 *	1.99 (1.14, 3.48)	0.013 *	1.91 (1.04, 3.49)	0.033 *
Not meet PA + Lower Diet. Qual.	1.57 (1.17, 2.09)	0.002 *	1.76 (1.15, 2.69)	0.008 *	1.46 (0.87, 2.45)	0.144
**Adults 65 years and over**						
PA or Diet. Qual.						
Total PA MET-minutes/week-per increase of 100 point	0.99 (0.99, 1.00)	0.017 *	1.00 (0.99, 1.00)	0.151	0.98 (0.98, 0.99)	<0.001 *
Diet. Qual. (HEI-2015)—per increase of 10 point	1.03 (0.94, 1.14)	0.512	1.02 (0.87, 1.19)	0.811	1.02 (0.92, 1.14)	0.645
PA + Dietary Quality						
Met PA + Higher Diet. Qual.	Ref	-	Ref	-	Ref	-
Met PA + Lower Diet. Qual.	0.90 (0.60, 1.34)	0.6	0.98 (0.52, 1.86)	0.951	0.82 (0.46, 1.47)	0.498
Not meet PA + Higher Diet. Qual.	1.28 (0.87, 1.89)	0.2	1.41 (0.87, 2.29)	0.159	1.25 (0.70, 2.23)	0.438
Not meet PA + Lower Diet. Qual.	1.11 (0.76, 1.62)	0.59	1.09 (0.64, 1.86)	0.736	1.19 (0.74, 1.92)	0.471

Note: Odds ratio (95% CI) and *p*-values were obtained by performing multiple logistic regression adjusted for sex, age, race/ethnicity, education, poverty income ratio and weight status. PA = physical activity; MET = metabolic equivalent of task; HEI = Healthy Eating Index. Met PA recommendations is PA time ≥ 600 MET-minutes/week; higher dietary quality was defined as 60.1 < HEI-2015 ≤ 99.6 (3rd tertile); lower dietary quality was defined as 10 ≤ HEI-2015 ≤ 60.1 (1st and 2nd tertiles). * *p* < 0.05.

## Data Availability

The present study used data from: (1) Centers for Disease Control website: https://wwwn.cdc.gov/nchs/nhanes/sasviewer.aspx; and (2) U.S. Department of Agriculture website: https://www.ars.usda.gov/northeast-area/beltsville-md-bhnrc/beltsville-human-nutrition-research-center/food-surveys-research-group/docs/fped-data-tables.
